# Suicidal Ideation Following a Single Dose of Melatonin in a Patient With Major Depressive Disorder: A Case Report

**DOI:** 10.1002/ccr3.71984

**Published:** 2026-02-02

**Authors:** Rahim Badrfam, Atefeh Zandifar

**Affiliations:** ^1^ Community Mental Health Center (CMHCs) Alborz University of Medical Sciences Karaj Alborz Iran; ^2^ Non‐Communicable Diseases Research Center Alborz University of Medical Sciences Karaj Alborz Iran; ^3^ Department of Psychosomatic Medicine, Shariati Hospital Alborz University of Medical Sciences Karaj Alborz Iran; ^4^ Social Determinants of Health Research Center Alborz University of Medical Sciences Karaj Alborz Iran; ^5^ Clinical Research Development Unit, Department of Psychiatry, Imam Hossein Medical Education Center Alborz University of Medical Sciences Karaj Alborz Iran

**Keywords:** depression, insomnia, melatonin, suicide

## Abstract

Melatonin is a neurohormone that primarily regulates sleep patterns and circadian rhythms by interacting with melatonin receptors MT1 and MT2. Disruptions in circadian rhythms may contribute to mood disorders; for example, individuals with major depressive disorder often have significantly lower melatonin levels compared to healthy individuals. In this report, we present a patient who experienced a depressed mood and nighttime sleep disturbances and was treated with melatonin. In patients with depression, factors such as insomnia or poor psychosocial conditions can increase the risk of suicidal thoughts.

## Key Clinical Message

In patients with depression, it is essential to conduct a comprehensive assessment of suicide risk, especially when insomnia or psychosocial stressors are present.

## 1 Introduction

Melatonin is a neurohormone that primarily regulates sleep patterns and circadian rhythms through pathways mediated by melatonin receptors 1 (MT1) and 2 (MT2) [1]. Some studies suggest that melatonin has limited direct effects on promoting sleep. Its primary action may be related to signaling the suprachiasmatic nucleus (SCN) that it is nighttime, thereby enhancing light synchronization and stabilizing the sleep/wake cycle [2].

Melatonin administration, typically at doses ranging from 2 to 10 mg taken 1 to 2 h before bedtime, is often used to treat insomnia symptoms, including those associated with mood and neurocognitive disorders [3]. Previous research indicates that dysregulation of circadian rhythms may contribute to mood disorders, with patients suffering from major depressive disorder exhibiting significantly lower melatonin levels compared to healthy individuals [4].

In this report, we present a case of a patient with major depressive disorder who experienced recent nocturnal sleep disturbances and developed sudden suicidal thoughts after receiving their first dose of melatonin.

## 2 Case History/Examination

The patient is a 40‐year‐old married man with a secondary education and no prior psychiatric hospitalizations. He works as a food distributor. Approximately 4 months ago, he experienced a significant financial loss due to a failed investment in a construction project. About a month ago, he began to develop symptoms of depression, including a low mood, decreased appetite, sleep difficulties characterized by late‐onset insomnia, occasional restlessness, reduced concentration, and diminished social interaction. Notably, he has not had any suicidal thoughts or desires. The patient reports that after eventually falling asleep, he can sleep continuously for 7–8 h without issues. Due to the worsening of his symptoms, he consulted a psychiatrist, who prescribed fluoxetine capsules (10 mg daily) and melatonin tablets (3 mg, to be taken 2 h before bedtime). The patient had no recent history of taking psychiatric medications or herbal remedies.

Since the psychiatrist's appointment was scheduled for the late afternoon, the patient took the first dose of melatonin at bedtime. Because acute fluoxetine treatment may cause initial sleep disturbances [5], the patient decided, based on the psychiatrist's recommendation, to postpone starting fluoxetine until the next morning. Four hours after taking a dose of melatonin, he woke up in the middle of the night at 2 AM, feeling agitated and confused. In a sudden moment of distress, he contemplated jumping from the fourth‐floor balcony of their apartment in an attempt to end his life. However, he ultimately refrained from doing so, worried that it would frighten his wife and child. This was particularly notable, as the patient had no prior history of suicidal thoughts or attempts.

The patient's history reveals that he had experienced a depressive episode at the age of 20, triggered by a decline in academic performance due to a lack of interest in his studies, leading him to leave education and enter the workforce. During that time, he was treated with a short course of antidepressants, including fluoxetine and propranolol, which resulted in clinical improvement. There is no personal or family history of suicidal thoughts or attempts, nor is there any history of psychiatric disorders in his family. The patient is currently free of any significant physical illnesses and is not taking any other medications. About 6 months ago, his routine tests yielded normal results without any issues. Additionally, he has no history of smoking, alcohol, or substance use.

## 3 Differential Diagnosis, Investigations, and Treatment

The following morning, he and his wife returned to the psychiatrist at the Community‐Based Mental Health Center (CMHCs) to discuss his distressing episode. After assessing the situation, the psychiatrist deemed it necessary for the patient to undergo emergency psychiatric hospitalization due to ongoing suicidal thoughts and referred him to a university inpatient center for further evaluation. It was also advised that he discontinue all medications until he could be assessed thoroughly. A review of the patient's medication history confirmed that only one melatonin tablet had been prescribed. The only factor that could be seen as a potential trigger for an acute exacerbation of the patient's mood was the use of a single dose of melatonin. The psychiatrist approached this conclusion with caution, as there were no similar reports in the literature. Therefore, discontinuing the medication was prioritized as the first course of action. Blood levels of melatonin and fluoxetine were not measured due to financial constraints. Additionally, it was determined that repeated administration of melatonin would not be included in this patient's treatment plan to ensure safety, and alternative medications to improve sleep quality would be utilized.

## 4 Conclusion and Results (Outcome and Follow‐Up)

After the patient was hospitalized, he was closely monitored and prescribed olanzapine at a dose of 5 mg to help improve his mood and address his sleep issues. This medication led to a noticeable improvement in his mood symptoms, and he experienced restful sleep the following night. The next day, although he still felt low and had feelings of hopelessness, he did not express any suicidal thoughts. The treatment plan continued with olanzapine, alongside psychoeducation and daily psychiatric visits. On the fifth day of his hospitalization, the patient requested to be discharged. Despite the psychiatrist's insistence on the importance of continuing inpatient treatment, the patient was discharged with the consent of his caregivers.

Upon follow‐up at an outpatient visit the following week, the patient showed some improvement in his mood and did not report any suicidal thoughts. He was then prescribed olanzapine and fluoxetine, starting at 10 mg daily and increasing to 20 mg daily 1 week later. Over time, his mood continued to improve. One month after discharge, the patient remained in a stable mood; however, he continued to report periods of exacerbation of mood symptoms without suicidal thoughts, primarily due to escalating financial problems from decreased job performance.

Three months after his admission, the patient demonstrated significant improvements in both personal and occupational functioning. His sleep patterns were adequate, and a plan was established to gradually reduce the dosage of his medications. Olanzapine was initially reduced to 2.5 mg daily and then discontinued. Five months after the exacerbation of symptoms and the initiation of treatment, there was a significant improvement in the patient's mood as well as in his personal and social functioning. The patient returned to their full‐time job, and the fluoxetine dose was reduced to 10 mg daily before being discontinued after a short period.

## 5 Discussion

The coincidence of starting melatonin and the sudden development of suicidal thoughts in the patient underscores the need for careful monitoring of melatonin's effects, especially in individuals with a history of mood disorders. This situation is particularly alarming because the patient did not experience any suicidal thoughts during the month of depressive symptoms before beginning melatonin. It is also important to consider that other factors, such as fluctuations in depressive symptoms, anxiety‐related agitation, and stress responses, could potentially explain this case.

The patient was prescribed a dose of 3 mg of melatonin to be taken 2 h before bedtime. A review of randomized controlled trials suggests that melatonin use is associated with a gradual reduction in sleep onset latency and an increase in total sleep time, with the most effective results observed at a dose of 4 mg per day [6], which is similar to the dosage used in this patient.

Recent research has focused on the association between insomnia medications and suicidality [7]. For instance, a longitudinal cohort study conducted in Denmark from 2007 to 2016 found that individuals treated with melatonin had a four‐fold higher rate of suicide and a five‐fold higher rate of suicide attempts compared to those who were not treated with melatonin [8]. The authors of this study stressed the importance of being vigilant about suicide risk, particularly in individuals with coexisting mental health conditions who are using melatonin. They attributed the observed association between melatonin treatments and suicidal behavior primarily to the potential mediating effects of psychiatric and sleep disorders in these patients.

Additionally, a case report involving a 43‐year‐old woman with primary insomnia and a family history of suicide noted that the addition of ramelteon—a novel melatonin MT1 and MT2 receptor subtypes agonist that has specific effects on melatonin receptors in the suprachiasmatic nucleus [9]—to her treatment with flunitrazepam was associated with the emergence of disinhibition, aggression, and ultimately suicidal attempts. These symptoms rapidly resolved following the discontinuation of ramelteon [10]. The authors suggested that competitive metabolism by CYP3A4 between flunitrazepam and ramelteon, leading to elevated levels of both drugs, may have contributed to these adverse effects although they acknowledged that this could have been coincidental.

In another population‐based cohort study with different output results in Sweden involving young people aged 6–18 years, researchers observed a reduced risk of intentional self‐harm among women with depression and anxiety after initiating melatonin treatment. This improvement was noted 12 months after starting the medication, compared to the month prior, when they were not on melatonin. The authors highlighted the mediating role of sleep interventions in this outcome [11]. The differences in results between this study and similar studies, including our reported case, may be attributed to the one‐year cohort design of the latter study, as well as the inclusion of participants from the pre‐adult age group in a population where over 50% had co‐morbidity with Attention Deficit Hyperactivity Disorder (ADHD). There is a significant relationship between sleep disorders and underlying ADHD in children and adolescents. Additionally, these sleep disorders are common side effects of ADHD medications, which may have influenced the final results of this study [12].

In the case being discussed, the patient's pre‐existing mood disorder and recent history of insomnia could potentially explain the emergence of suicidal thoughts [13]. However, the sudden onset of these thoughts following just a single dose of melatonin is concerning. While this does not establish a definitive causal relationship, it is important to consider the possibility of such an association, especially given the risks associated with melatonin use.

Most of the positive evidence in the existing research comes from cohort studies following a course of melatonin treatment. This presentation is notable as it reports instances of suicidal ideation following a single dose of melatonin. Therefore, it is essential to be aware of the potential increased risk of suicide associated with melatonin, particularly in individuals with a recent history of depressive or mood disorders, and to provide appropriate guidance to clients regarding this risk.

In examining the potential link between a single dose of melatonin and the onset of suicidal thoughts in the patient in question, it is crucial to emphasize that this is merely a single case report, and no rechallenge with melatonin was conducted. Additionally, several confounding factors must be taken into account, including the patient's active major depressive disorder, recent insomnia, and significant psychosocial stressors. It is also important to highlight that a temporal association alone does not establish a cause‐and‐effect relationship. It is also important to emphasize that, although case study methodology is widely used in health research, the limited information it provides for causal inferences poses a limitation in evaluative studies [14].

## Author Contributions

**Rahim Badrfam:** conceptualization, investigation, project administration, resources, validation, visualization, writing – original draft, writing – review and editing. **Atefeh Zandifar:** conceptualization, investigation, project administration, resources, validation, visualization, writing – original draft, writing – review and editing.

## Funding

The authors have nothing to report.

## Ethics Statement

This case report meets the ethical guidelines.

## Conflicts of Interest

The authors declare no conflicts of interest.

## Data Availability

Data available on request due to privacy/ethical restrictions.
